# A Retrospective Analysis of Nasopharyngeal Biopsy Results in Adult Patients

**DOI:** 10.7759/cureus.13455

**Published:** 2021-02-20

**Authors:** Hacer Baran, Sedat Aydın, Elif Elibol

**Affiliations:** 1 ENT, Istanbul Lutfi Kırdar City Hospital, Istanbul, TUR; 2 Otolaryngology, Istanbul University Medical Faculty, Istanbul, TUR; 3 Otolaryngology, Ankara Yıldırım Beyazıt University, Yenimahalle Government Hospital, Ankara, TUR

**Keywords:** nasopharyngeal carcinoma, nasopharyngeal biopsy, nasopharyngeal neoplasm

## Abstract

Introduction

The widespread use of endoscopic evaluation in otolaryngology practice enables early diagnosis and treatment of nasopharyngeal cancers. This study aimed to evaluate the complaints, endoscopic examination findings, preferred radiological imaging methods, and pathology results of patients who underwent endoscopic nasopharyngeal biopsy.

Methods

Medical records of 956 patients (543 males and 413 females) who underwent nasopharyngeal biopsy between 2013 and 2018 were analyzed retrospectively. Age, gender, application complaint, endoscopic imaging findings, preferred radiological imaging methods, and biopsy results of the patients were retrospectively analyzed. The patients were grouped as benign and malignant according to their pathological diagnosis. Statistical evaluation was made between the benign and malignant groups in terms of parameters.

Results

The age range of the patients was 16-88 years. Pathology results of 102 patients were reported as malignancy. The most common malignancy was undifferentiated non-keratinized carcinoma (2.5%). The most frequently reported benign pathology was chronic nonspecific inflammation. The most common complaint was nasal congestion, which was observed at a rate of 63.1%. The most frequently requested radiological imaging was MRI with 43.1%.

Conclusion

The image of the masses found during endoscopic examination is very important to make a decision of malignancy. Even if the repeated biopsy is performed in patients with biopsy due to suspicious mass or with additional symptoms for nasopharyngeal carcinoma, radiological imaging is important for the exclusion of malignancy.

## Introduction

The nasopharynx (NP) is the upper part of the pharynx located on the soft palate at the posterior of the nasal cavity. Many malignant and benign tumors originate from this region [1]. Due to the regression of adenoid tissue in the adolescent period, mass monitoring in this region is important for malignancy in adulthood [2,3]. The use of nasopharyngoscopy in routine otorhinolaryngologic examination made early detection of any masses in this region more likely [4]. Patients with a nasopharyngeal mass may present the hospital with complaints such as hearing loss, nasal congestion, epistaxis, and mass in the neck. Unilateral or bilateral serous otitis can often be detected in examination findings, and in the endoscopic examination, a mass on the back wall of the nose can also be detected [4,5]. The gold standard for the detection of malignancy is nasopharyngeal biopsy taken under endoscopic imaging, although imaging methods such as magnetic resonance imaging (MRI) and computed tomography (CT) are used to monitor the nasopharyngeal region and to detect the masses in this region [6].

This study aimed to evaluate the distribution of the biopsy results of patients who presented to the ENT outpatient clinic and had a mass in the nasopharyngeal region or who were suspected of NP malignancy due to symptoms. We also evaluated the rates of repeated biopsy and which radiological tests were required.

## Materials and methods

A total of 956 patients who presented to our ENT outpatient clinic between the years 2013 and 2018, diagnosed with a nasopharyngeal mass after endoscopic examination, and underwent nasopharyngeal biopsy were included in the study. After ethical approval from the Institutional Review Board, patient records were accessed for retrospective data collection. In the patient's records, age, gender, application complaint, examination findings, image of the mass in endoscopic examination, and requested imaging methods were retrospectively scanned. Pathology results were categorized into benign and malignant. These groups were also divided into subgroups, with benign masses further categorized into chronic nasopharyngitis, reactive lymphoid hyperplasia, Thornwaldt cyst, necrotized granulomatous inflammation, and other, and malignant masses categorized into undifferentiated nonkeratinized carcinoma, differentiated nonkeratinized carcinoma, lymphoma, keratinized squamous cell carcinoma, and other. Patients under the age of 16 years, those whose material was reported as insufficient after biopsy, and those with insufficient patient records were excluded from the study.

Statistical analysis

Statistical analysis of the data was conducted using IBM SPSS for Windows Version 22.0 (IBM Corp., Armonk, NY, USA). Statistical evaluation was made between the benign and malignant groups in terms of the following parameters: gender, age, admission complaints, physical examination findings, radiological examinations, and whether repeated biopsy was required. Quantitative variables were expressed as mean ± standard deviation (SD) and range values, whereas categorical variables were expressed as number (n) and percentage (%). The differences between malignant and benign groups were examined using the chi-square test. A p-value of <0.05 value was considered statistically significant.

## Results

A total of 956 patients (543 males and 413 females) participated in the study. The age range of those included in the study was determined to be between 16 and 88 years, and the mean age was found to be 41.9 years. When the patients were grouped according to their pathological results, 854 patients were included in the benign group, while the results of 102 patients were reported to be malignant (Table 1). The most common presenting complaint was nasal obstruction (63.1%). On physical examination, the most common symptom was unilateral or bilateral chronic effusion (48.1%). If the physical examination findings and complaints of the patient on endoscopic examination suggested nasopharyngeal malignancy, radiological imaging methods were also requested in the diagnosis. When the radiological imaging methods requested for nasopharyngeal evaluation were examined, it was identified that nasopharyngeal MRI was requested for 412 (43.1%) patients and CT was requested for 132 (13.8%) patients. Generally, we preferred MRI to evaluate soft tissue, while CT was preferred in patients who could not be evaluated with MRI or who were thought to have additional sinus pathology. At follow-up, we did not need to repeat the biopsy for 629 (65.8%) patients; it was repeated once for 264 (27.6%) patients, twice for 48 (5%) patients, and three times for 15 (1.6%) patients.

**Table 1:** Distribution of patients according to pathology results SCC, squamous cell carcinoma

Pathology results	N	Percentage
Benign		
Chronic nasopharyngitis	592	61.9
Reactive lymphoid hyperplasia	229	24
Thornwaldt cyst	12	1.3
Necrotized granulomatous inflammation	8	0.8
Other	13	1.3
Total	854	89.3
Malignant		
Undifferentiated carcinoma nonkeratinized	44	4.6
Differentiated nonkeratinized SCC	24	2.5
lymphoma	23	2.4
Keratinized SCC	6	0.6
Other	5	0.5
Total	102	10.7

When the benign and malignant groups were compared in terms of gender, there was no statistically significant difference (p = 0.538). Of the patients whose pathological results were reported as malignant, 44 were female and 58 were male (female-to-male ratio: 1:3). Of the results reported as benign, 369 were female and 485 were male. When the benign and malignant groups were compared in terms of age, it was found that the patients in the benign group were statistically significantly younger than the patients in the malignant group (p = 0.000). The average age of patients in the malignant group was found to be 54.46 years, while the average age of patients in the benign group was 40.5 years.

Comparison of complaints and symptoms between benign and malignant groups are given in Table 2. When the groups were compared in terms of the radiological examinations taken, it was seen that nasopharyngeal MRI was requested from a total of 412 patients. It was determined that 83 patients in the malignant group were asked for MRI due to suspicious mass, and 329 patients in the benign group were evaluated with MRI. The rate of MRI requests from malignant patients was found to be statistically significantly higher than that of benign patients (p = 0.00). A total of 132 patients were asked for CT, of whom 128 were found to be benign and 4 were malignant. It was found that a significantly higher rate of CT was requested from the patients in the benign group (p = 0.001).

**Table 2:** Comparison of complaints and symptoms between benign and malignant groups

	Benign		Malignant		p-Value
	n	%	n	%	
Complaints					
Nasal congestion	518	85.9	85	14.1	0.000
Aural fullness	468	89.1	57	10.9	0.46
Epistaxis	21	51.2	20	48.8	0.000
Symptoms					
Effused otitis	414	90.2	45	9.8	0.233
Mass in the neck	118	60.2	78	39.8	0.000
Smooth surfaced mass	640	98.9	7	1.1	0.000
Cystic mass	30	96.8	1	3.2	0.138
Suspicious mass (irregular-looking, bleeding mass )	50	36	89	64	0.000
No mass	93	94.9	5	5.1	0.035

It was observed that 264 patients out of 327 patients underwent re-biopsy after the first biopsy. It was found that 264 of these patients underwent biopsy one time, 48 of them twice, and 15 of them underwent biopsy three times. There was no statistically significant difference between the groups in terms of repeating the biopsy (p = 0.540). Of 102 patients in the malignant group, 18 required biopsy once, and it was found that malignancy was diagnosed at the second time in nine patients and at the third time in four patients.

## Discussion

The NP is an irregular cuboidal structure located under the base of the skull behind the nasal cavity, and its mucosa is lined with nonkeratinized columnar ciliated epithelium with areas of common squamous metaplasia. Subepithelial connective tissue is rich in lymphoid cells [7]. The masses in this region are important for adults in terms of malignancy. The symptoms of nasopharyngeal malignancy are not specific, and, additionally, due to its localization, patients generally present in the late period, and this causes delay in treatment [6,8].

In order to differentiate malignancy from lymphoid tissue, outside of radiological evaluation methods, several different screening and examination methods such as evaluation of antibodies of the Epstein-Barr virus and smear sampling have been described in the literature. However, in the diagnosis of nasopharyngeal malignancy, biopsy accompanied by nasopharyngoscopy is the gold standard [5,9-10].

Among the complaints of patients presenting with NP mass, nonspecific findings such as nasal congestion, hearing loss, aural fullness, neck mass, and nose bleeding can be included [4,5]. In the literature, the most common complaint was nasal congestion [11,12]. In our study also, the most common complaint was nasal congestion. When the complaints of the patients were examined, a statistically significant difference was found between the groups in terms of nasal congestion, epistaxis, and mass in the neck. This indicates that when the mass is detected, in order to distinguish benign or malignant, more attention should be paid to investigate the malignant mass if nasal congestion, epistaxis, and mass in the neck are observed along.

There was no difference between the groups in terms of obstruction and fullness in the ear. In addition, in our study, 57 (10.9%) patients with malignant disease had complaints of aural fullness. In the study by Glynn et al, malignancy was identified in nasopharyngeal biopsy in 4.7% of adults with conductive hearing loss secondary to serous otitis media. A comprehensive examination of the NP should be performed with fiber optic nasal endoscopy in any adult who was diagnosed with serous otitis or who was admitted with aural fullness. All masses within the NP should be correctly identified and biopsies should be performed for each mass, giving priority to the suspected masses. If the appearance of the NP is normal in adults with serous otitis media, it may be preferable to follow closely instead of biopsy [13].

In the study of King et al., the sensitivity of the endoscopic examination findings was found to be 90% [4]. In our study, pathological outcome was reported as malignant in 89 (64%) of 139 patients who were evaluated by endoscopic examination and followed up for suspicion of malignancy. Biopsy results from an irregular surfaced fragile mass were more common in the malignant group. In the benign group, the smooth surfaced mass and the cystic mass were significantly more frequent.

Endoscopic nasopharyngeal biopsy can be performed under general or local anesthesia. In the study of Waldron et al., a comparison was made between local and general anesthesia, and no statistically significant difference was found between the two. In this study, there is no information about patient comfort between the two groups [14]. Endoscopic biopsies of the patients in our study were performed under local anesthesia, within an office setting, from two or three different suspicious areas. However, there were patients who underwent biopsy for the second and third time. Regarding biopsy repeat rates, Arslan et al. reported a 51.6% secondary biopsy necessity in diagnosing malignancy in their study [6]. In our study, a subsequent biopsy was needed in 31 (30.4%) of 102 patients. These results show that if there is a suspicion of malignancy in endoscopic examination findings and current clinical findings, the biopsy should be performed again, even if the pathology result is reported as benign.

In patients who are evaluated due to mass in the NP, besides biopsy, radiological evaluation methods such as MRI and CT can be used. Regarding this subject, in the study of King et al., 95% accuracy rate, 100% sensitive, 93% specificity, and 87% positive predictive value were determined for nasopharyngeal MRII in primary nasopharyngeal cancers. Bercin et al. stated that the sensitivity of MRI was 88.2% in nasopharyngeal malignancies [4,8]. In our study, MRI was also found to be the most preferred imaging method. In patients diagnosed with NP carcinoma, it was observed that MRI was requested at the rate of 81.3%. In addition, it was determined that in patients whose pathology results were reported as benign, MRI was requested at the rate of 38.5%. It was observed that the MRI rate was higher than that in other studies. A reason for this MRI rate, which was higher than other studies, could be the request of MRI before the diagnosis of malignancy instead of invasive procedure in our clinic and the request of MRI before secondary biopsy from patients whose first results were reported as benign.

In the studies by Arslan et al. and Bercin et al., the malignancy rate was 4.6% and 2.9%, respectively [6,8]. In our study, the malignancy rate was found to be 10.7%. The reason for the rate of malignancy being higher from other studies could be the request for nasopharyngeal MRI in cases where patients who are evaluated for nasopharyngeal mass are suspected of malignancy.

Male predominance has been generally described in patients diagnosed with NP malignancy. In our study, the male-to-female ratio was found to be 1:3. Patients diagnosed with malignancy in patients who underwent nasopharyngeal biopsy in various studies were also older than benign patients [6,8,11]. In our study, a similar relationship was found between the benign and malignant groups. The average age of patients in the malignant group was found to be 54.46 years, while the average age of patients in the benign group was 40.5 years (p < 0.005).

According to the results of biopsies of 98 patients who did not have a mass appearance, malignancy rate was found to be 5.1%. Serous otitis was the most common complaint in these patients. In patients with long-term serous otitis without mass, we thought that planning for biopsy after nasopharyngeal MRI may be significant in terms of evaluating submucosal disease, performing a biopsy in the suspicious localization, and reducing the rate of repeating the biopsy procedure.

In our study, regarding the findings of endoscopic examination, 1.1% malignancy was detected as a result of biopsy of smooth surfaced masses. If there is no suspicious mass appearance in patients who presented to our clinic with serous otitis complaint, it may be important to prefer MRI instead of an invasive procedure in terms of malignancy exclusion in order to avoid an additional invasive procedure. If there is a suspicious mass in the endoscopic examination, a biopsy is required. This study has several limitations, as the samples were retrospectively small and the study was performed in single-center design.

## Conclusions

Nasopharyngeal cancers are located in an anatomical region that can be diagnosed later in life in terms of localization and symptomatology. Adult patients with nasal congestion, ear congestion, hearing loss, and swelling in the neck should be evaluated endoscopically in the outpatient clinic, and biopsy should be performed for the patients with a suspected mass in the NP.

## References

[1] Prasad ML, Perez-Ordonez B (2009). Nonsquamous lesions of the nasal cavity, paranasal sinuses and nasopharynx. Diagnostic Surgical Pathology of the Head and Neck. 2nd Edition.

[2] Kamel RH, Ishak EA (1990). Enlarged adenoid and adenoidectomy in adults: endoscopic approach and histopathological study. J Laryngol Otol.

[3] Wang WH, Lin YC, Weng HH, Lee KF (2011). Narrow-band imaging for diagnosing adenoid hypertrophy in adults: a simplified grading and histologic correlation. Laryngoscope.

[4] King AD, Vlantis AC, Bhatia KS (2011). Primary nasopharyngeal carcinoma: diagnostic accuracy of MR imaging versus that of endoscopy and endoscopic biopsy. Radiology.

[5] Ni XG, Zhang QQ, Wang GQ (2016). Classification of nasopharyngeal microvessels detected by narrow band imaging endoscopy and its role in the diagnosis of nasopharyngeal carcinoma. Acta Otolaryngol.

[6] Arslan N, Tuzuner A, Koycu A, Dursun S, Hucumenoglu S (2018). The role of nasopharyngeal examination and biopsy in the diagnosis of malignant diseases. Braz J Otorhinolaryngol.

[7] Engin K, Erişen L (2003). Baş ve Boyun kanserleri. Baş ve Boyun kanserleri. Nobel Tıp Kitapevi.

[8] Bercin S, Yalciner G, Muderris T, Gul F, Deger HM, Kiris M (2017). Pathologic evaluation of routine nasopharynx punch biopsy in the adult population: is it really necessary?. Clin Exp Otorhinolaryngol.

[9] Lee AW, Ng WT, Chan YH, Sze H, Chan C, Lam TH (2012). The battle against nasopharyngeal cancer. Radiother Oncol.

[10] Ng RH, Ngan R, Wei WI, Gullane PJ, Phillips J (2014). Trans-oral brush biopsies and quantitative PCR for EBV DNA detection and screening of nasopharyngeal carcinoma. Otolaryngol Head Neck Surg.

[11] Berkiten G, Kumral TL, Yildirim G, Uyar Y, Atar Y, Salturk Z (2014). Eight years of clinical findings and biopsy results of nasopharyngeal pathologies in 1647 adult patients: a retrospective study. B ENT.

[12] Bilici S, Yıldız M, Gokduman AR, Yigit O (2017). Clinical appearances in patients with nasopharyngeal malignancy: retrospective analysis. KBB Forum.

[13] Glynn F, Keogh IJ, Ali TA, Timon CI, Donnelly M (2006). Routine nasopharyngeal biopsy in adults presenting with isolated serous otitis media: is it justified?. J Laryngol Otol.

[14] Waldron J, Van Hasselt CA, Wong KY (1992). Sensitivity of biopsy using local anesthesia in detecting nasopharyngeal carcinoma. Head Neck.

